# Integrated metabolomic, transcriptomic and network analysis elucidates therapeutic mechanisms of *Ganoderma lucidum* spore oil against granulomatous pulmonary nodules

**DOI:** 10.3389/fphar.2025.1612043

**Published:** 2025-06-10

**Authors:** Ya Liu, Wendong Xu, Kexin Wang, Zhiye You, Xiaohong Chen, Huihui Ti, Xiaoli Liang, Lin Cao, Hongfei Cai, Juyan Liu, Zifeng Yang

**Affiliations:** ^1^ The First Affiliated Hospital of Guangzhou Medical University, Guangzhou Institute of Respiratory Health, Guangzhou, China; ^2^ National Engineering Research Center of Pharmaceutical Processing Technology of Traditional Chinese Medicine and Drug Innovation, Guangdong Provincial Key Laboratory of Medicinal Lipid, Guangzhou, China; ^3^ Guangzhou National Laboratory, Guangzhou, China; ^4^ School of Chinese Materia Medica, Guangdong Pharmaceutical University, Guangzhou, China; ^5^ KingMed School of Laboratory Medicine, Guangzhou Medical University, Guangzhou, China; ^6^ Engineering Technology Research Center of Intelligent Diagnosis for Infectious Diseases in Guangdong Province, Guangzhou Medical University, Guangzhou, China

**Keywords:** granulomatous pulmonary nodule, *G. lucidum* spore oil, metabolic balance, inflammatory responses, network analysis

## Abstract

Granulomatous pulmonary nodules represent a substantial health threat, with no available targeted therapies. Interventions based on medicinal plants approaches are pivotal for prevention and treatment. *Ganoderma lucidum* (GL) is a traditional botanical drug to treat inflammatory ailments. The spores of this fungus demonstrate distinct influences on immune response regulation, arthritis, and malignant growth inhibition. However, there is a lack of research on its application in the treatment of granulomatous pulmonary nodules. This study investigated the therapeutic potential and mechanism of *G. lucidum* spores (GLS) in mice with granulomatous pulmonary nodules. Utilizing granulomatous pulmonary nodules mice model induced by a peptide from the mycobacterial soda protein, we comprehensively evaluated the anti-granulomatous pulmonary nodules effects of GLS oil (GLSO) *in vivo* through various indices, such as lung micro-CT analysis, pathological conditions, inflammatory cytokine levels, chemokine. Metabolomics, transcriptomics, and network analysis were employed to elucidate its mechanisms. Results revealed that GLSO markedly reduced granuloma area and ground glass opacities in the lungs while attenuating pulmonary inflammation and chemokine levels. Metabolomic analysis identified 14 differential metabolites regulated by GLSO, including those involved in alanine, aspartate, and glutamate metabolism, arginine biosynthesis, and the tricarboxylic acid cycle. Transcriptomic and network analysis pinpointed the PI3K-Akt-mTOR signaling pathway as a central mechanism of GLSO action. Integrated analyses further indicated that GLSO alleviates granulomatous pulmonary nodules by inhibiting p-AKT and p-mTOR activation, thereby restoring metabolic balance and reducing inflammatory responses and chemokine secretion. These results position GLSO as a promising therapeutic candidate for granulomatous pulmonary nodules, acting through multifaceted regulatory mechanisms.

## 1 Introduction

In recent years, the widespread use of low-dose computed tomography (CT) scans and increased public health awareness have significantly enhanced the detection of asymptomatic pulmonary nodules (Hendrix et al., 2023). The detection rate of pulmonary nodules by CT is approximately 79.79%, with at least 95% of these being benign, predominantly granulomas or intrapulmonary lymph nodes (Mazzone and Lam, 2022). Currently, no standardized approach exists for managing detected pulmonary nodules (Christensen et al., 2024). While some pulmonary nodules represent precancerous lesions of lung cancer, early screening and intervention can provide critical opportunities for early detection and treatment of malignant tumors (Mankidy et al., 2023). However, for those nodules where the benign or malignant nature is indeterminate (El Alam, Byrne and Hammer, 2023), unnecessary biopsies or blind surgeries may lead to avoidable physical and psychological harm to patients (Gould et al., 2023). Both domestic and international guidelines recommend imaging follow-up for incidentally discovered solitary pulmonary nodules, but this approach is insufficient to meet current clinical demands, highlighting the urgent need for effective intervention strategies (Farjah et al., 2022).

*Ganoderma lucidum*, a traditional medicinal fungus with a history of use spanning thousands of years, has garnered attention for its health benefits (Swallah et al., 2023; He et al., 2023). Its spores, tiny oval reproductive cells released during the fungus’s maturation, contain all the genetic material of the organism (Xu and Li, 2019). Rich in fatty acids and polysaccharides, *G. lucidum* spores have been extensively studied for their therapeutic properties (Liang et al., 2019). However, due to their robust double-walled structure, the spores are challenging to absorb when consumed whole (Qi et al., 2024). *Ganoderma lucidum* spore oil (GLSO), extracted through supercritical CO_2_ low-temperature methods from broken-wall *G. lucidum* spore powder (Zheng et al., 2024; Liu et al., 2024), retains most of the lipid components, including unsaturated fatty acids and triglycerides (Liu et al., 2024). Recent research has demonstrated GLSO’s efficacy in inhibiting inflammation in a collagen-induced rheumatoid arthritis mouse model (Heo et al., 2023). Through comprehensive microbiome and metabolomic analysis, Wu et al. revealed the immune-enhancing properties of GLSO in mice (Wu et al., 2020), while Zhang et al. identified GLSO as a novel antioxidant capable of extending the lifespan of fruit flies (Zhang et al., 2021). Further studies have shown that GLSO potentiates the effects of cyclophosphamide by inhibiting programmed cell death protein-1 (PD-1), thereby prolonging the survival of H22 tumor-bearing mice (Jiang et al., 2024). Despite these promising findings, there is a lack of research on the application of GLSO in the treatment of granulomatous pulmonary nodules.

The advent of multi-omics technologies and the continuous evolution of virtual computing have propelled the development of network analysis, grounded in systems biology, offering novel strategies for exploring therapeutic mechanisms (Hopkins, 2008). Metabolomics enables the monitoring of endogenous small-molecule metabolite changes within organisms. By assessing the overall metabolic shifts under normal physiological conditions, in disease states, and during drug interventions, it provides valuable insights into disease onset and the mechanisms of therapeutic action (Qiu et al., 2023). Transcriptomics captures gene expression data, allowing for the genomic-level interpretation of drug mechanisms (Dopazo, 2014). Network analysis integrates diverse biological datasets, including gene expression and metabolomics data (Hu et al., 2022; Zhao et al., 2024), to more accurately predict drug action mechanisms, thereby providing a scientific framework for understanding the efficacy of complex biological systems.

In this study, mice with granulomatous pulmonary nodules induced by a peptide from the mycobacterial soda protein were utilized. The therapeutic effects of GLSO on pulmonary nodules were assessed through pulmonary CT imaging, lung tissue pathology, and measurement of inflammatory and chemotactic factors in lung tissues. A comprehensive approach integrating metabolomics, transcriptomics, and network analysis was then employed to explore the potential therapeutic mechanisms of GLSO. This study aims to elucidate the effects and mechanisms of GLSO in treating granulomatous pulmonary nodules, thereby laying the groundwork for clinical application and providing experimental insights for future therapeutic strategies.

## 2 Materials and methods

### 2.1 Materials and reagents

GLSO was manufactured from Guangzhou Hanfang Pharmaceutical Co., Ltd. (batch 2408013, approval number G20100150). Freund’s incomplete adjuvant and Sepharose beads were obtained from Sigma-Aldrich. The RNA extraction kit was purchased from Beyotime (Shanghai, China). Antibodies were sourced from Akt (STARTER, S0B688), p-AKT (Proteintech, 66444-1-IG), mTOR (Proteintech, 66888-1-IG), p-mTOR (Proteintech, 67778-1-IG).

### 2.2 Animal experimental design

C57BL/6J mice (16–18 g) were obtained from Zhuhai BesTest Bio-Tech Co., Ltd. and housed in the Guangzhou Laboratory Animal Centre under specific pathogen-free conditions, maintained at a constant temperature of 24°C–25°C with a 12-h light/dark cycle. Each group contained eight mice, with *ad libitum* access to food and water.

Mice were randomly assigned to four groups: control group (soybean oil, Control), model group (soybean oil, Model), GLSO-L group (0.3 g/kg GLSO, GLSO-L), and GLSO-H group (1.2 g/kg GLSO, GLSO-H). Treatment was administered continuously for 51 days. On day 30, model group was anesthetized with tribromoethanol and subcutaneously injected with 50 µg of soda and 0.25 mL of incomplete Freund’s adjuvant. On day 44, 6000 agarose beads conjugated with soda were injected into the tail vein of the mice. On day 50, thoracic imaging was performed using Micro-CT. On day 51, the lung, heart, liver, spleen, and kidney were excised and weighed, with the lungs frozen for subsequent analysis.

### 2.3 Micro-CT imaging of mouse lungs

Granulomatous pulmonary nodule formation was assessed using micro-CT imaging on day 50. Mice from Control, Model, GLSO-L, and GLSO-H groups were lightly anesthetized with isoflurane to ensure stable respiration during scanning, with each mouse remaining under anesthesia for approximately 5 min. Imaging was performed on the PerkinElmer Quantum GX micro-CT system under the following conditions: tube voltage of 90 kV, X-ray tube current of 88 μA, and scan duration of 4 min (Mecozzi et al., 2020).

### 2.4 Histopathological assessment of lung granulomatous areas

Lung tissues were fixed in 4% paraformaldehyde for 24 h to preserve tissue structure. Following fixation, tissues were rinsed in phosphate-buffered saline (PBS) three times (5 min each) and dehydrated through a graded ethanol series: 70% ethanol for 1 h, 80% for 1 h, 95% for 1 h, and 100% ethanol twice, 1 h each. Tissues were then cleared in xylene twice, 20 min each before paraffin embedding at 60°C for 2 h. Embedded tissues were sectioned into 4 μm-thick slices using a rotary microtome (Leica RM2235), and mounted onto poly-L-lysine–coated glass slides. For staining, sections were deparaffinized in xylene twice, 10 min each, then rehydrated in descending ethanol concentrations: 100% ethanol twice for 5 min, 95% for 5 min, 80% for 5 min, and 70% for 5 min, followed by a rinse in distilled water for 5 min. Hematoxylin staining was performed for 8 min, after which sections were rinsed under running tap water for 10 min to allow for bluing. Eosin staining was then carried out for 2 min, followed by rapid dehydration in 95% ethanol (2 min) and 100% ethanol twice (2 min each). Sections were cleared in xylene twice (5 min each) and coverslipped using a neutral resinous mounting medium. Pathological changes were observed under a microscope, and images were scanned for further analysis. The extent of granuloma formation was quantitatively assessed by measuring the granulomatous areas.

### 2.5 Quantitative real-time PCR analysis of inflammatory cytokines and chemokines

Total RNA was extracted from tissue samples using Beyotime RNA extraction reagent. The concentration and purity of the extracted RNA were assessed using a NanoDrop 2000 spectrophotometer (Thermo Fisher Scientific, United States). Complementary DNA (cDNA) was synthesized using the Beyotime cDNA reverse transcription kit. Quantitative real-time PCR (qRT-PCR) was performed with gene-specific primers and SYBR Green on a thermal cycler. The gene-specific primers are as follows.

**Table:** 

Gene	Forward primer (5′-3′)	Reverse primer (5′-3′)
IL-1β	TGG​ACC​TTC​CAG​GAT​GAG​GAC​A	GTT​CAT​CTC​GGA​GCC​TGT​AGT​G
TNF-α	GGT​GCC​TAT​GTC​TCA​GCC​TCT​T	GCC​ATA​GAA​CTG​ATG​AGA​GGG​AG
IFN-γ	CAG​CAA​CAG​CAA​GGC​GAA​AAA​GG	TTT​CCG​CTT​CCT​GAG​GCT​GGA​T
IFN-β	GCC​TTT​GCC​ATC​CAA​GAG​ATG​C	ACA​CTG​TCT​GCT​GGT​GGA​GTT​C
CXCL9	CCT​AGT​GAT​AAG​GAA​TGC​ACG​ATG	CTA​GGC​AGG​TTT​GAT​CTC​CGT​TC
CXCL10	ATC​ATC​CCT​GCG​AGC​CTA​TCC​T	GAC​CTT​TTT​TGG​CTA​AAC​GCT​TTC
CCL2	GCT​ACA​AGA​GGA​TCA​CCA​GCA​G	GTC​TGG​ACC​CAT​TCC​TTC​TTG​G
CCL4	ACC​CTC​CCA​CTT​CCT​GCT​GTT​T	CTG​TCT​GCC​TCT​TTT​GGT​CAG​G

### 2.6 Transcriptomic analysis of mice lung

The RNA sequencing and analysis were carried out by Genedenovo Biotechnology Co., Ltd. (Guangzhou, China). The details are as follows: total RNA was extracted from mouse lung tissues using the RNA Extraction Kit (Beyotime, Shanghai). Poly(A)-tailed mRNA was isolated using oligo-dT magnetic beads, followed by fragmentation and reverse transcription into cDNA with random primers to construct double-stranded cDNA libraries. Sequencing adapters were ligated to both ends of the cDNA fragments, followed by PCR amplification and library purification. The quality of the libraries was assessed using the Agilent 2100 Bioanalyzer to ensure appropriate fragment size and concentration. Qualified libraries were sequenced on the Illumina NovaSeq 6000 platform using paired-end 150 bp (PE150) sequencing. Differential gene expression analysis was performed with DESeq2 software, and subsequent Gene Ontology (GO) and Kyoto Encyclopedia of Genes and Genomes (KEGG) functional enrichment analyses were conducted.

### 2.7 Metabolomics analysis of mice lung

Approximately 20 mg of lung tissue was placed in a grinding tube containing 750 µL of 80% methanol-water and homogenized under cooling conditions (4°C). The mixture was centrifuged at 13,000 rpm for 15 min to collect the supernatant, with the extraction repeated once. The supernatants were combined and vacuum-dried at 35°C for 4 h to remove residual solvents. The dried residue was resuspended in 600 µL of phosphate buffer (0.2 M Na_2_HPO_4_, 0.2 M NaH_2_PO_4_, pH 7.4) containing 0.05% TSP (sodium trimethylsilyl propionate-d_4_) as an internal standard. After centrifugation at 13,000 rpm for 10 min, the clarified supernatant was transferred to a 5 mm NMR tube. The ^1^H-NMR spectra were recorded on a Bruker AVNEO 600 MHz NMR spectrometer (Karlsruhe, Germany) at 25°C using the NOESY pulse sequence for water signal suppression. Acquisition parameters included 78 scans, 82,468 data points, and a spectral width of 8 kHz. The resulting spectra were processed using Chenomx NMR Suite software for Fourier transformation, phase adjustment, baseline correction, and chemical shift calibration.

### 2.8 Western blotting

Lung tissues were ground after 200 uL of lysis buffer (Beyotime, China) supplemented with protease and phosphatase inhibitor cocktail and then homogenates were incubated on ice for 30 min. Next the concentrations of protein were quantified using the BCA Protein Assay Kit (Beyotime, Shanghai). Each sample was adjusted to the same protein concentration, combined with 5× SDS loading buffer, and then boiled at 95°C for 10 min to denature the proteins. Proteins were extracted from lung tissue, and concentrations were quantified using the BCA Protein Assay Kit (Beyotime, Shanghai). The extracted proteins were separated by SDS-PAGE and transferred onto PVDF membranes. Membranes were blocked with 5% skimmed milk at room temperature for 1 h to minimize non-specific binding. They were then incubated overnight at 4°C with primary antibodies targeting Akt, p-Akt, mTOR, p-mTOR, and GAPDH. The following day, membranes were incubated with HRP-conjugated secondary antibodies for 1 h at room temperature. Protein bands were visualized using a chemiluminescent substrate and captured for analysis. Protein bands were visualized using ECL detection reagents (Thermo Fisher, United States) and imaged using a chemiluminescence imaging system (Thermo Fisher iBrightFL1500, United States). Band intensity was quantified using ImageJ software and normalized to the expression of GAPDH or β-actin as a loading control.

### 2.9 Statistical analysis

Data were analyzed using two-way analysis of variance (ANOVA) with Tukey’s *post hoc* test for multiple comparisons, and Student’s t-test was applied to compare two groups. p-values were adjusted using the Benjamini–Hochberg false discovery rate. Statistical significance was set at P < 0.05.

## 3 Results

### 3.1 Identification of GLSO components for quality control

**FIGURE 1:**
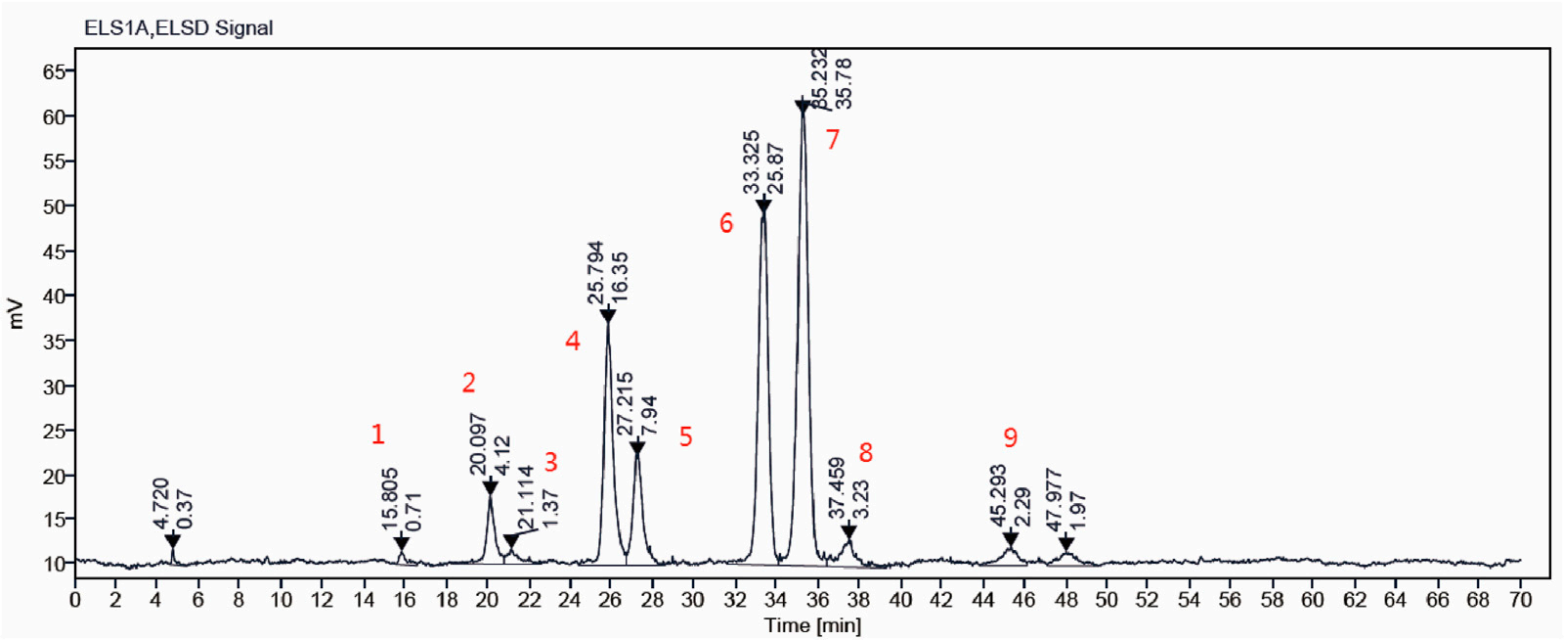
The main components of GLSO samples were determined by HPLC-ELSD.

The composition of GLSO was detected using high performance liquid chromatography with evaporative light scattering detection (HPLC-ELSD) (Figure 1A). Nine main triglycerides were identified as the primary constituents of GLSO by comparing the retention time with the standard substances (Supplementary Table S1; Supplementary Figure S1). The total content of these triglycerides in GLSO was 76.04%, and the two most abundant components in GLSO, triolein and 1,2-dioleoyl-3-palmitin, were quantified as 20.45% and 27.12%, respectively.

### 3.2 Safety profile of GLSO in the treatment of granulomatous pulmonary nodule

**FIGURE 2:**
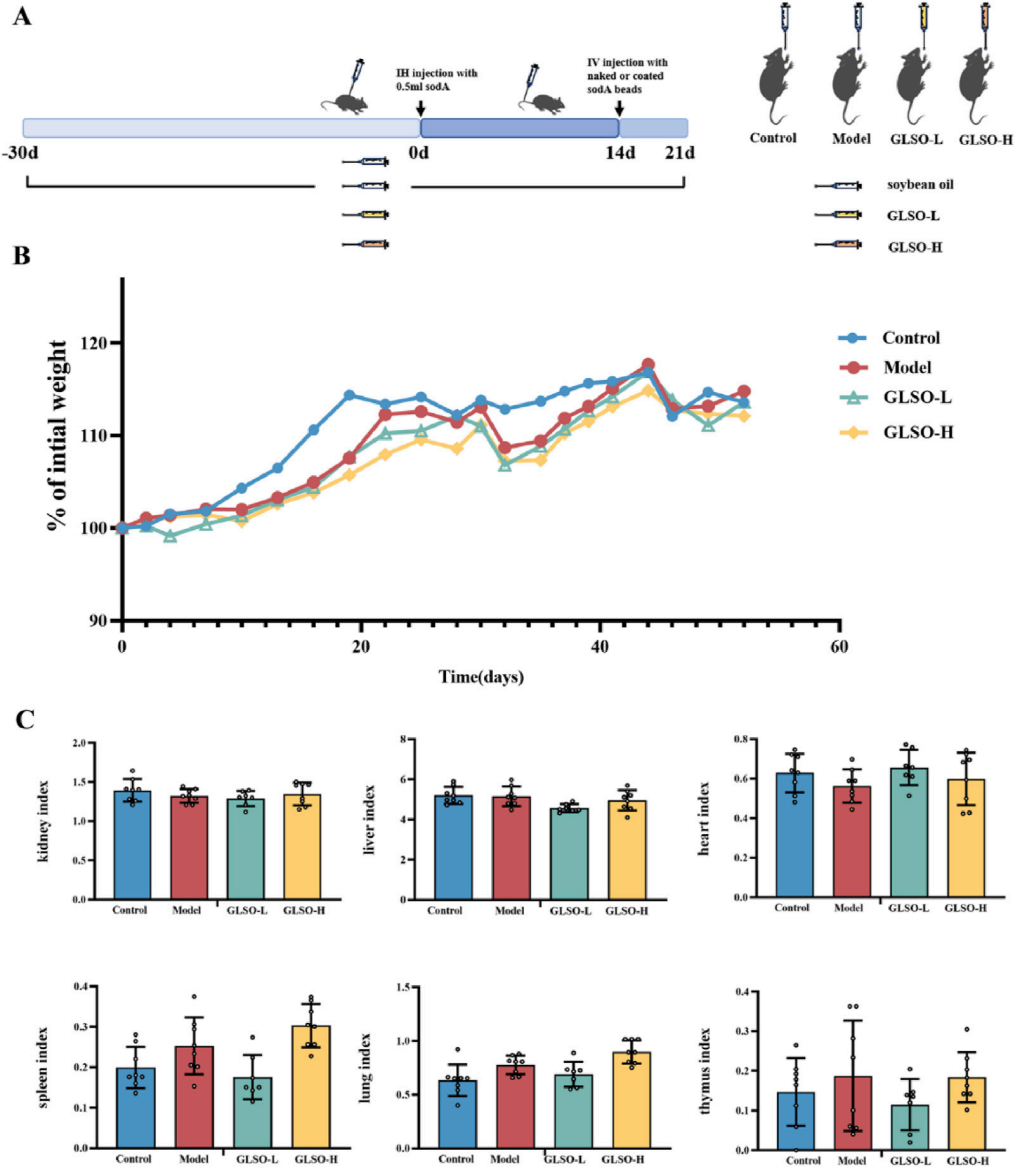
The safety profile of GLSO. Schematic summary of animal experiment **(A)**, body weight gain **(B)**, organ index **(C)**, n = 8 per group.

A granulomatous pulmonary nodule model was established in C57BL/6J mice *via* subcutaneous injection of soda peptide and related factors to evaluate the therapeutic effects of GLSO on lung granulomatous inflammation. After 51 days of GLSO administration, body weight and organ index measurements were conducted to assess the safety of GLSO (Figure 2A). Body weight analysis indicated a decrease in weight in all groups 1 day after the induction of sensitization by soda, with gradual recovery thereafter. The model and GLSO-treated groups exhibited decreased body weights compared to the control group, but no significant differences were observed (Figure 2B). Organ index measurements revealed no significant changes in the heart, liver, spleen, lung, thymus, and kidney indices in GLSO-L and GLSO-H-treated mice compared to the control group (Figure 2C).

### 3.3 Effect of GLSO on lung micro-CT and lung histopathology

**FIGURE 3:**
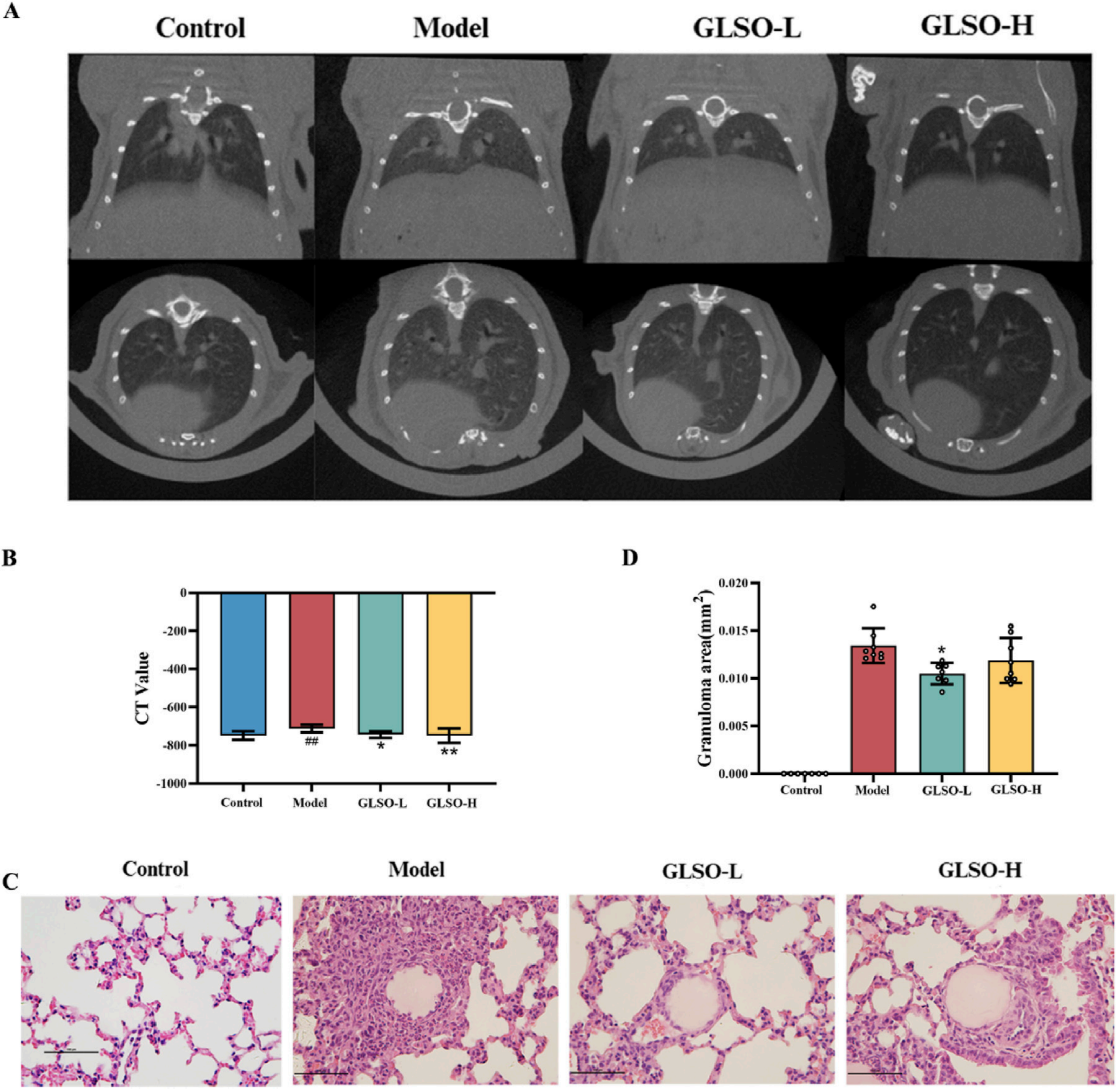
Effect of GLSO on lung micro-CT and lung histopathology. CT scanning imaging of the lungs **(A)** and CT values **(B)**, H&E staining images of lung tissue **(C)** and the statistics of granuloma area **(D)**. ^##^ *P* < 0.01 compared with the control group, ^*^ *P* < 0.05 and ^**^ *P* < 0.01 compared with the model group.

Lung imaging showed that the model group developed gray ground-glass shadows in comparison to the control group (Figure 3A). Statistical analysis of CT values revealed a significant increase in the CT value of the model group compared to the control group (P < 0.01) (Figure 3B). Ground-glass shadows were reduced in both GLSO-L and GLSO-H groups (Figure 3A). The CT values in GLSO-L and GLSO-H groups were significantly lower than those in the model group, with statistical significance (Figure 3B). Histological examination revealed numerous granulomas surrounding the agarose beads in the lung tissue of the model group (Figure 3C). In contrast, the area of epithelioid granulomas was significantly reduced in both GLSO-L and GLSO-H groups compared to the model group (Figure 3C). Statistical analysis confirmed a significant difference in granuloma area between the GLSO-L group and the model group (P < 0.05) (Figure 3D).

### 3.4 Effect of GLSO on inflammatory factors and chemokines

**FIGURE 4:**
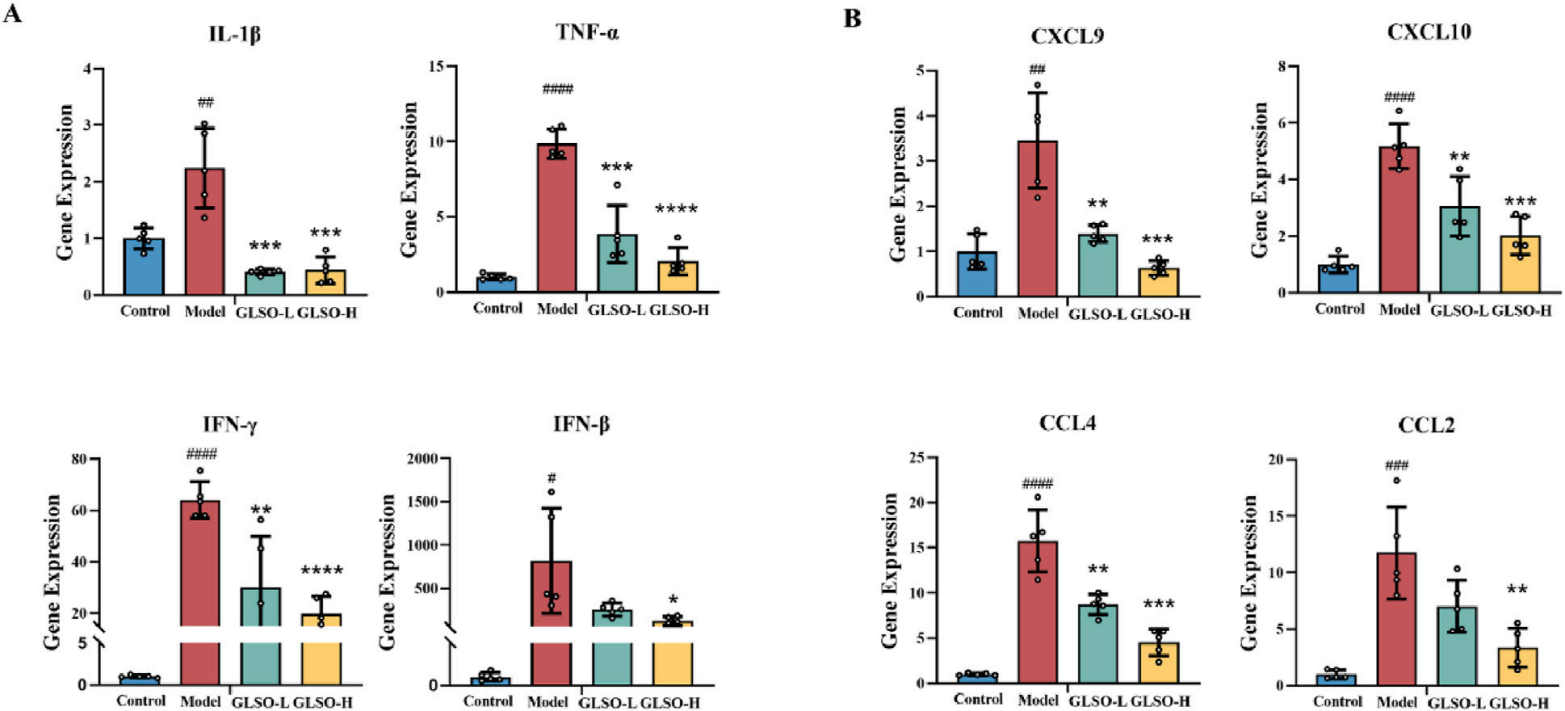
Effect of GLSO on inflammatory factors and chemokines. The mRNA expression levels of IL-1β, TNF-α, IFN-γ, and IFN-β **(A)**. The mRNA expression levels of CXCL9, CXCL10, CCL4, and CCL2 **(B)**. ^#^ *P* < 0.05, ^##^ *P* < 0.01, ^###^ *P* < 0.001, and ^####^ *P* < 0.0001, compared with the control group, ^*^ *P* < 0.05, ^**^ *P* < 0.01, ^***^ *P* < 0.001, and ^****^ *P* < 0.0001 compared with the model group.

Transcription levels of IFN-β, IFN-γ, IL-1β, and TNF-α serve as indicators of the inflammatory status in granulomatous pulmonary nodules of mice. Results revealed that, relative to the control group, expression of IFN-β, IFN-γ, IL-1β, and TNF-α was markedly elevated in the model group (Figure 4A). In contrast, GLSO-L and GLSO-H treatments led to significant reductions in IFN-γ, IL-1β, and TNF-α levels, with a notable decrease in IFN-β levels observed only in the GLSO-H group (Figure 4A). Chemokines primarily function to modulate the migration and localization of immune cells, and their transcriptional expression reflects granuloma formation. Compared to the control group, CCL2, CXCL10, CCL4, and CXCL9 levels were significantly higher in the model group (Figure 4B). Treatment with GLSO-L and GLSO-H resulted in significant reductions in CXCL10, CCL4, and CXCL9, with a marked decrease in CCL2 levels in the GLSO-H group (Figure 4B). These results suggest that GLSO effectively mitigates lung granulomatous inflammation in the mouse model.

### 3.5 Metabolic dysbiosis is ameliorated in mice with granulomatous pulmonary nodule after GLSO treatment

**FIGURE 5:**
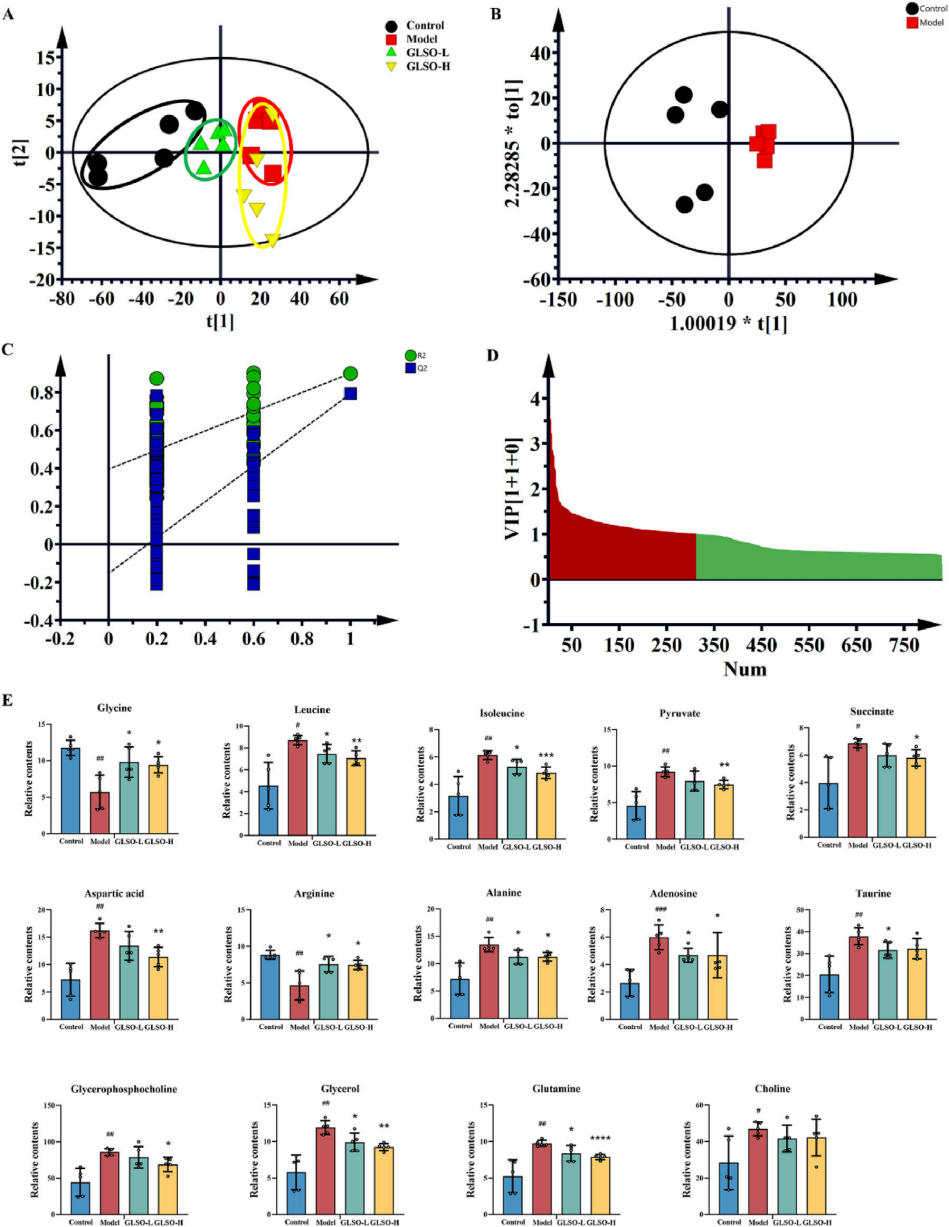
The metabolic homeostasis of GLSO. Scatter plot of principal component analysis **(A)**, OPLS-DA scores plots from control group compared with model group **(B)**, PLS-DA corresponding validation plot of permutation test for 200 times from control group compared with model group **(C)**, Variable importance in projection (VIP) plot from control group compared with model group **(D)**, Differential metabolites **(E)**, ^#^ *P* < 0.05, ^##^ *P* < 0.01, ^###^ *P* < 0.001, compared with the control group, ^*^ *P* < 0.05, ^**^ *P* < 0.01, ^***^ *P* < 0.001, and ^****^ *P* < 0.0001 compared with the model group.

To elucidate the mechanisms underlying the therapeutic effects of GLSO in granulomatous pulmonary nodule treatment, metabolic profiling of lung samples was performed. Principal component analysis (PCA) revealed a distinct separation between the control and model groups, confirming the successful establishment of the model. Moreover, the GLSO-treated groups showed a trend toward the control group, suggesting a restorative effect of GLSO (Figure 5A). To further visualize group differences, orthogonal partial least squares discriminant analysis (OPLS-DA) was applied (Figure 5B). This supervised pattern recognition method necessitates external model validation, which was confirmed by the regression analysis shown in Figure 5C. The relatively large slopes of the regression lines and the small intercepts between the lower regression line and the vertical axis, with *R* ^2^ and Q^2^ values lower than the original model, indicate effective validation of the model. Using the OPLS-DA model, differential metabolites were identified with VIP >1 (Figure 5D) and P < 0.05, leading to the identification of 14 metabolites associated with granulomatous pulmonary nodules.

Compared to the control group, levels of glutamine, choline, aspartic acid, isoleucine, leucine, alanine, pyruvate, succinic acid, glycerophosphocholine, taurine, glycerol, and adenosine were elevated in the model group, while arginine and glycine levels were reduced (Figure 5E). Following GLSO intervention, 13 metabolites were significantly restored. Specifically, the GLSO-L group exhibited a significant decrease in leucine, isoleucine, alanine, taurine, glycerol, glutamine, and adenosine, while arginine and glycine levels significantly increased. In the GLSO-H group, glutamine, isoleucine, leucine, alanine, pyruvic acid, succinic acid, glycerophosphate, glycerol, and aspartic acid significantly decreased, while arginine and glycine levels significantly increased.

**FIGURE 6:**
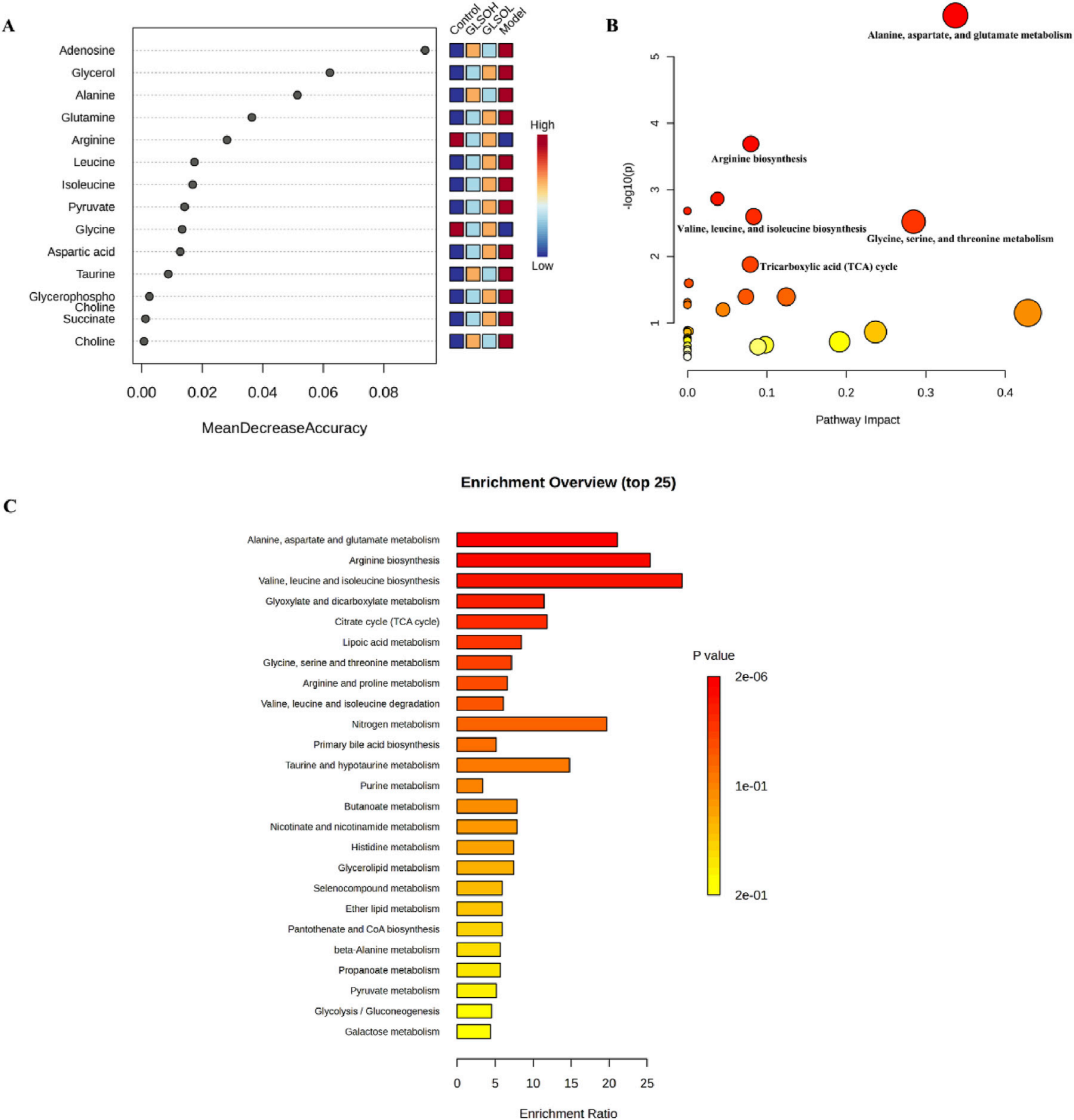
The metabolic pathway analysis of GLSO. Most important metabolites in random forests **(A)**. Features ranked by their contributions to classification accuracy. The enrichment of metabolic pathway **(B,C)**.

Machine learning analysis was applied to identify key metabolites regulated by GLSO in the treatment of granulomatous pulmonary nodule. Adenosine, glycerol, and alanine were identified as pivotal metabolites (Figure 6A). Metabolic pathway enrichment analysis of the differentially regulated metabolites in the GLSO groups revealed significant alterations in pathways such as alanine, aspartate, and glutamate metabolism; arginine biosynthesis; valine, leucine, and isoleucine biosynthesis; glycine, serine, and threonine metabolism; and the tricarboxylic acid (TCA) cycle (Figures 6B,C).

### 3.6 Transcriptomic regulation of the lung by GLSO in granulomatous pulmonary nodule mice

**FIGURE 7:**
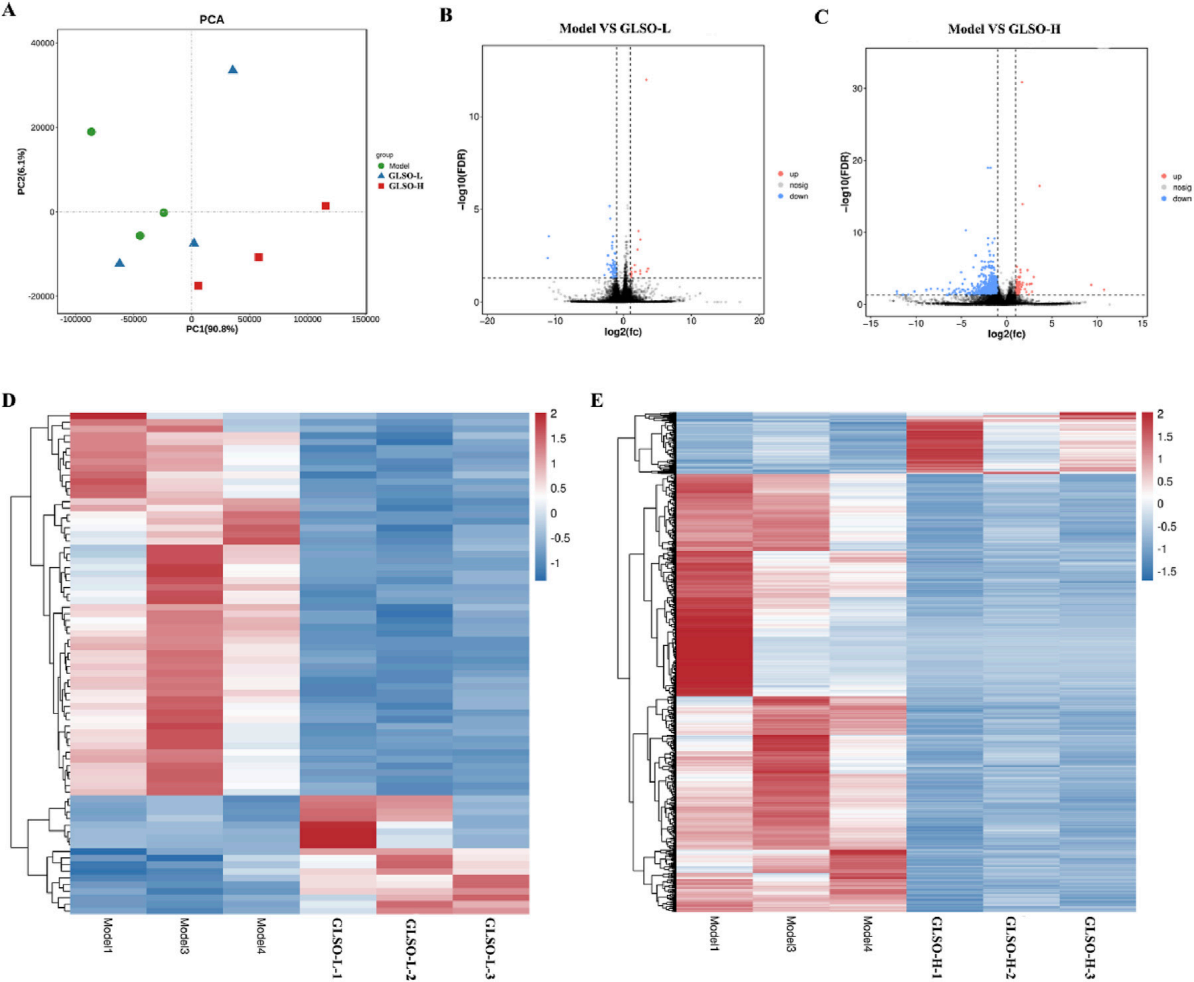
Differentially expressed genes in granulomatous pulmonary nodules mice. Scatter plot of principal component analysis **(A)**, Volcano map of DEGs **(B,C)**. Heatmap of DEGs **(D,E)**.

Transcriptome sequencing was performed to investigate the mechanisms by which GLSO modulates granulomatous pulmonary nodules in mice. PCA analysis revealed that the GLSO-H group was more distantly clustered from the model group compared to the GLSO-L group (Figure 7A). In the GLSO-L group, 76 differentially expressed genes (DEGs) were identified in lung tissue, with 18 genes significantly upregulated and 58 significantly downregulated compared to the model group (Figures 7B,D). In the GLSO-H group, 916 genes were significantly regulated, with 113 upregulated and 803 downregulated relative to the model group (Figures 7C,E).

### 3.7 Correlation analysis between lung metabolomic and transcriptomic analyses

**FIGURE 8:**
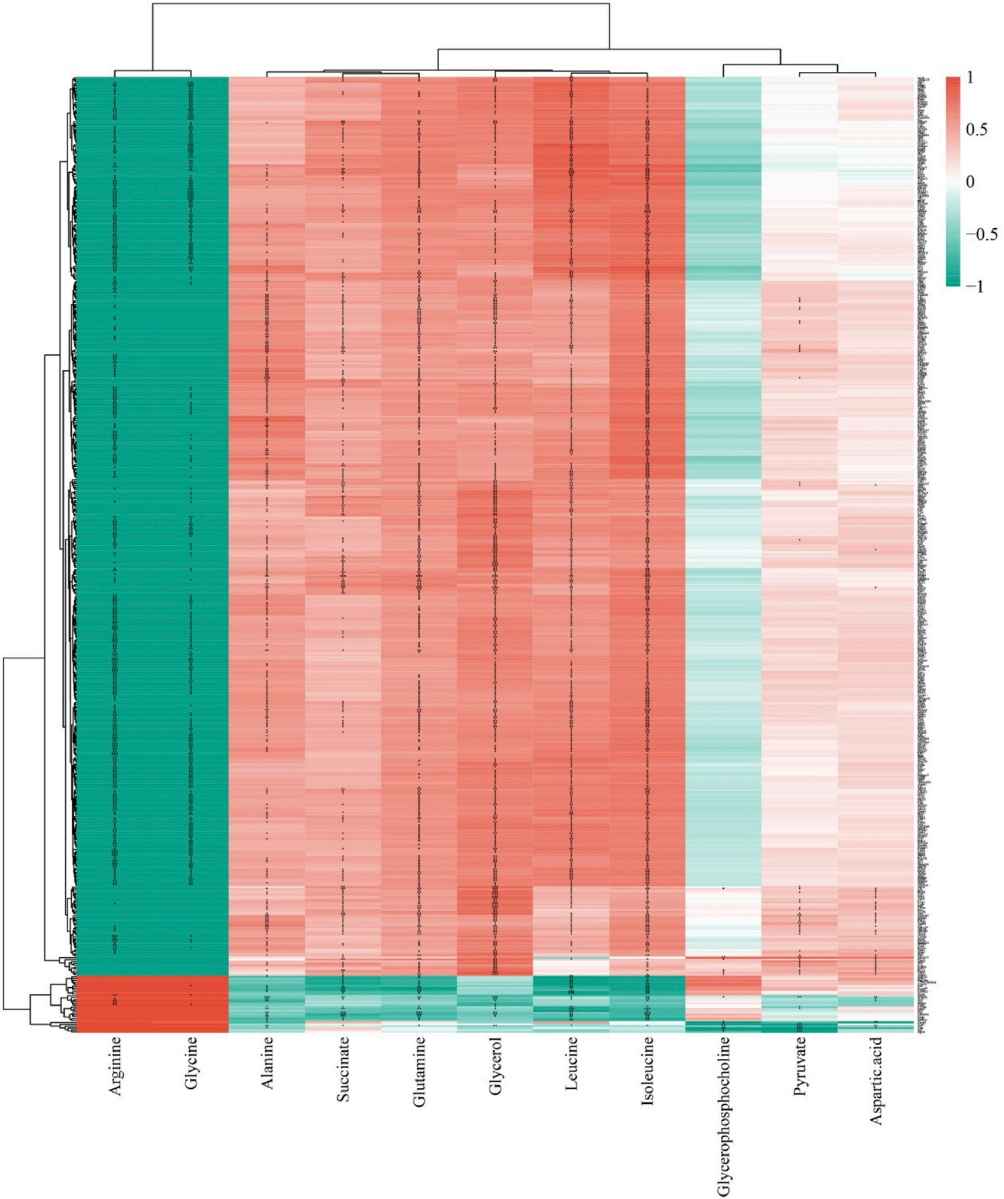
The correlation analysis of differential metabolites and DEGs regulated by GLSO.

A correlation analysis between 14 differential metabolites and 926 DEGs regulated by GLSO revealed that 11 metabolites were significantly correlated with 503 DEGs (P < 0.001, correlation |r| > 0.8). Of these, 605 metabolite-gene pairs exhibited positive correlations, while 425 pairs showed negative correlations (Figure 8). The correlation coefficient and P value of the 1030 metabolite-gene pairs was shown in Supplementary Table S2.

### 3.8 Analysis of key DEGs of GLSO regulation based on network analysis

**FIGURE 9:**
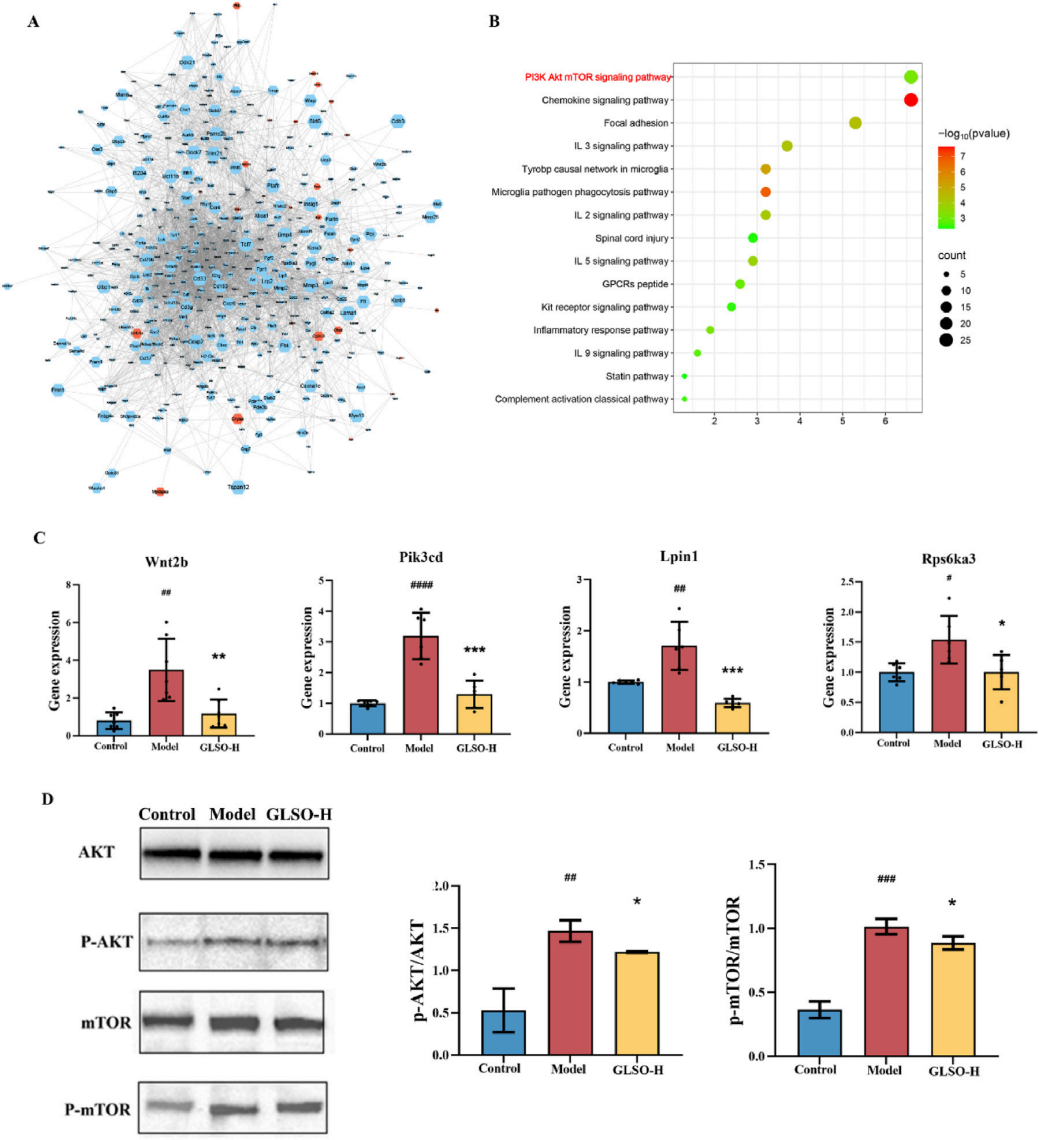
Network analysis and experiment validation. Gene-gene interaction network **(A)**, KEGG pathway enrichment analysis results **(B)**, the gene and protein level of PI3K-Akt-mTOR pathway **(C, D)**.

The 927 DEGs regulated by GLSO were analyzed using STRING to construct a protein-protein interaction (PPI) network. The network comprised 763 nodes and 4709 edges, which were visualized using Cytoscape software. NetworkAnalyzer was applied to calculate closeness centrality, radiality, and R values, with a median R value of 0.54, to identify key DEGs. After sorting, 441 key DEGs were selected, with upregulated genes depicted in orange and downregulated genes in blue (Figure 9A). The 441 genes significantly regulated by GLSO were further analyzed for KEGG pathway enrichment using DAVID, resulting in the identification of 15 significantly enriched pathways (P < 0.05, q < 0.05) (Figure 9B). Notably, the chemokine signaling pathway, inflammatory factor signaling pathway, and PI3K-Akt-mTOR pathway were prominently regulated, with the PI3K-Akt-mTOR pathway exhibiting the highest number of counts and the lowest P value (Figure 9B).

### 3.9 GLSO inhibited the AKT/mTOR signaling in granulomatous pulmonary nodule mice

To further investigate the involvement of the PI3K-Akt-mTOR pathway, four DEGs (Wnt2b, Pik3cd, Lpin1, and Rps6ka3) were validated. As shown in Figure 9C, the mRNA levels of these genes were significantly elevated in the lung tissue of mice with granulomatous pulmonary nodules compared to the control group. However, treatment with GLSO-H significantly reduced the mRNA expression of Wnt2b, Pik3cd, Lpin1, and Rps6ka3 in the lung tissue. qRT-PCR results confirmed that these findings were consistent with the transcriptome data. To further assess the impact of GLSO-H on the PI3K-Akt-mTOR pathway, Western blotting analysis was performed on key proteins involved in the pathway. As depicted in Figure 9D, the phosphorylation levels in the lung tissues of model mice were significantly higher than those of control mice, while GLSO-H treatment notably inhibited this phosphorylation. These findings indicate that GLSO-H effectively suppresses the activation of the PI3K-Akt-mTOR signaling pathway in granulomatous pulmonary nodule tissue.

## 4 Discussion

Although inflammatory pulmonary nodules are typically benign, they can still pose significant health risks. These nodules can lead to persistent inflammation in lung tissue, disrupt the structural integrity of the lung parenchyma (Akçiçek et al., 2024), and impair normal ventilation, resulting in symptoms such as cough, expectoration, shortness of breath, and dyspnea. This, in turn, decreases patients' quality of life. Furthermore, prolonged inflammatory stimulation can increase the likelihood of malignant transformation of these nodules, thereby elevating the risk of lung cancer (Rice et al., 2019; Shen et al., 2022). Currently, treatments such as glucocorticoids, immunosuppressants, and antibiotics are commonly employed (Sacco, Gazzin, Notarangelo and Delmonte, 2023; Smits et al., 2023), but these medications are associated with numerous side effects, and their long-term use can compromise both immune and metabolic functions (Newton, Leigh and Giembycz, 2010). Additionally, emerging treatments such as targeted therapy and immunotherapy are under investigation but are not yet widely available in clinical practice (Nakamizo et al., 2023).

This study reveals that GLSO significantly reduces the gray ground-glass opacities in lung images of mice with granulomatous pulmonary nodules, decreases the granuloma area, and lowers the levels of inflammatory mediators (IFN-β, IFN-γ, IL-1β, TNF-α) and chemokines (CCL2, CXCL10, CCL4, CXCL9), demonstrating its therapeutic potential for granulomatous pulmonary nodule. IFN-β, secreted by immune cells, enhances immune cell functions, particularly macrophages. Persistent elevation of IFN-β in chronic inflammation may contribute to the persistence of inflammation. IFN-γ, a T-cell-derived cytokine, regulates macrophage activity, and its high transcription level serves as an indicator of inflammation (Jorgovanovic, Song, Wang and Zhang, 2020). IL-1β, primarily produced by nonspecific immune cells, plays a critical role in chronic inflammation (Mantovani, Dinarello, Molgora and Garlanda, 2019), while TNF-α, a key pro-inflammatory cytokine, recruits immune cells to exacerbate the inflammatory response (Balkwill, 2006). Thus, the transcription levels of IFN-β, IFN-γ, IL-1β, and TNF-α reflect the inflammatory status of granulomatous pulmonary nodules in mice. Chemokines, which regulate immune cell migration and localization, serve as indicators of granuloma formation. CCL2, a monocyte chemotactic protein-1, promotes monocyte migration and their differentiation into macrophages (Ni et al., 2023). CXCL10 and CCL4 facilitate the migration of Th1 and CD8^+^ T cells to the granuloma (Soong, Henard and Melby, 2012), while CXCL9 recruits CD8^+^ T cells, Th1 cells, and NK cells, amplifying the immune response in granuloma areas (Tokunaga et al., 2018).

Metabolism plays a critical role in the pathogenesis of granulomatous pulmonary nodules. At the cellular level, macrophages, as the primary component of granulomas, undergo metabolic reprogramming in response to pathogen invasion or foreign body stimulation (Wang et al., 2019), profoundly influencing granuloma formation and progression. The accumulation of pyruvate in the acidic environment promotes granuloma formation (Krausgruber et al., 2023), whereas GLSO intervention reduces pyruvate levels. Furthermore, previous studies have shown increased lipid accumulation in granuloma areas, suggesting that lipid metabolism disrupts granuloma formation (Wilson, Mayr and Weichhart, 2019), with GLSO significantly decreasing glycerol, choline phosphate, and glycerol levels in the model group. Downstream products of amino acid metabolism can directly modulate immune responses; for instance, arginine regulates early immune responses, and GLSO can modulate various amino acid metabolite levels following intervention.

In this study, the levels of p-AKT and p-mTOR proteins in the lung tissue of model mice were significantly higher than in control mice, indicating robust activation of the PI3K-Akt-mTOR signaling pathway during granulomatous pulmonary nodule formation. The PI3K/AKT/mTOR pathway, a key regulator of cellular homeostasis, is a critical driver of inflammatory pathogenesis in various diseases, influencing metabolism, cell cycle, and immune responses (Szwed, Kim and Jacinto, 2021; Chen et al., 2024). In addition, chemokine signaling and the PI3K/AKT pathway exhibit extensive crosstalk. Specifically, chemokines induce cell polarization via the PI3K/Akt signaling axis, which facilitates directional cell migration (Fang et al., 2022). Notably, the phosphorylation of p-AKT and p-mTOR in lung tissue of model mice was significantly inhibited following GLSO intervention, demonstrating that GLSO effectively targets the PI3K-Akt-mTOR pathway, inhibiting its activation. This provides a novel therapeutic target and strategy for treating granulomatous pulmonary nodules.

In addition, it is worth noting that the apparent discrepancy between pathological improvement in the low-dose group and the more pronounced molecular changes in the high-dose group may stem from fundamental differences in biological responses at varying dosages. The pathological improvements in low-dose group may reflect successful tissue-level reprogramming that isn't fully captured by whole-tissue transcriptomics/metabolomics. Conversely, high-dose treatment might induce stronger cell-autonomous molecular changes in surviving cells that dominate omics signatures but fail to translate into macroscopic tissue repair.

Earlier research on granulomatous pulmonary nodules has primarily focused on conventional anti-inflammatory drugs (e.g., corticosteroids) or broad-spectrum immunosuppressants, which often lead to adverse effects and incomplete resolution of granulomas. In contrast, GLSO not only reduces granuloma formation and pulmonary inflammation but also corrects metabolic disturbances, a feature rarely reported in prior studies. Our metabolomic and transcriptomic analyses reveal that GLSO uniquely regulates key pathways such as arginine biosynthesis and the TCA cycle, which are critical for granuloma maintenance and immune cell function.

## 5 Conclusion

In conclusion, this study demonstrates that GLSO may alleviate granulomatous pulmonary nodules by modulating the PI3K-Akt-mTOR signaling pathway, inhibiting p-AKT and p-mTOR activation, correcting metabolic disturbances, and reducing the release of inflammatory factors and chemokines. These findings offer new therapeutic targets and potential drug candidates for the clinical treatment of granulomatous pulmonary nodules, while also broadening the potential applications of GLSO.

## Data Availability

The original contributions presented in the study are included in the article/Supplementary Material, further inquiries can be directed to the corresponding authors.
