# Supplementary material for: Integrated metabolomic, transcriptomic and network analysis elucidates therapeutic mechanisms of Ganoderma lucidum spore oil against granulomatous pulmonary nodules
Source: Front Pharmacol. 2025 Jun 10;16:1612043. doi: 10.3389/fphar.2025.1612043 (PMC12185471; doi:10.3389/fphar.2025.1612043)
Supplement: Supplementary file 1 [file Supplementaryfile1.docx]

**Integrated metabolomic, transcriptomic and network analysis elucidates therapeutic mechanisms of *Ganoderma lucidum* spore oil against granulomatous pulmonary nodules**

Ya Liu ^a#^, Wendong Xu ^b^, Kexin Wang ^a,c#^, Zhiye You ^d#^, Xiaohong Chen ^a^, Huihui Ti ^d^, Xiaoli Liang ^e^, Lin Cao ^b^, Hongfei Cai ^b^, Juyan Liu ^b *^, Zifeng Yang ^a,^^c,f *^

a. The First Affiliated Hospital of Guangzhou Medical University, Guangzhou Institute of Respiratory Health, Guangzhou, 510230, China.

b. National Engineering Research Center of Pharmaceutical Processing Technology of Traditional Chinese Medicine and Drug Innovation, Guangdong Provincial Key Laboratory of Medicinal Lipid, Guangzhou, 510240, China.

c. Guangzhou National Laboratory, Guangzhou, 510230, China.

d. School of Chinese Materia Medica, Guangdong Pharmaceutical University, Guangzhou 510006, China.

e. KingMed School of Laboratory Medicine, Guangzhou Medical University, Guangzhou, 511436, China.

f. Engineering Technology Research Center of Intelligent Diagnosis for Infectious Diseases in Guangdong Province, Guangzhou, 511436, China

*** Corresponding Author:**

Zifeng Yang (jeffyah@163.com)

Juyan Liu (maoyk@byshf.com)

#These authors contributed to this research equally.

**Table S1 The retention time (RT) and content of individual triglyceride peaks.**

| **No.** | **Name** | **Content** | **RT/min** |
| --- | --- | --- | --- |
| 1 | Trilinolein (LLL) | 0.56% | 15.805 |
| 2 | 1,2-Dilinoleoyl-3-olein (LLO) | 3.26% | 20.097 |
| 3 | 1,2-Dilinoleoyl-3-palmitin (LLP) | 1.08% | 21.114 |
| 4 | 1,2-Dioleoyl-3-linolein (OOL) | 12.92% | 25.794 |
| 5 | 1-Palmitoyl-2-oleoyl-3-linolein (POL) | 6.28% | 27.215 |
| 6 | Triolein (OOO) | 20.45% | 33.325 |
| 7 | 1,2-Dioleoyl-3-palmitin (OOP) | 27.12% | 35.232 |
| 8 | 1,3-Dipalmitoyl-2-olein (POP) | 2.55% | 37.459 |
| 9 | 1,2-Oleoyl-3-stearin (OOS) | 1.81% | 45.293 |

**Fig S1：The chromatograms of each standard substance are presented below.**

1. **LLL**

**
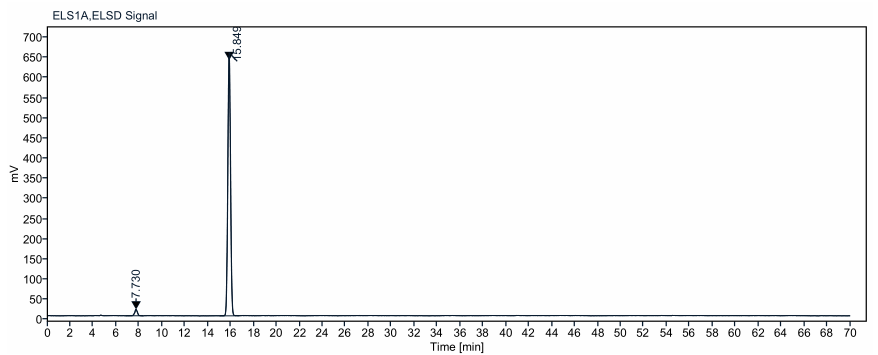
**

1. **LLO**

**
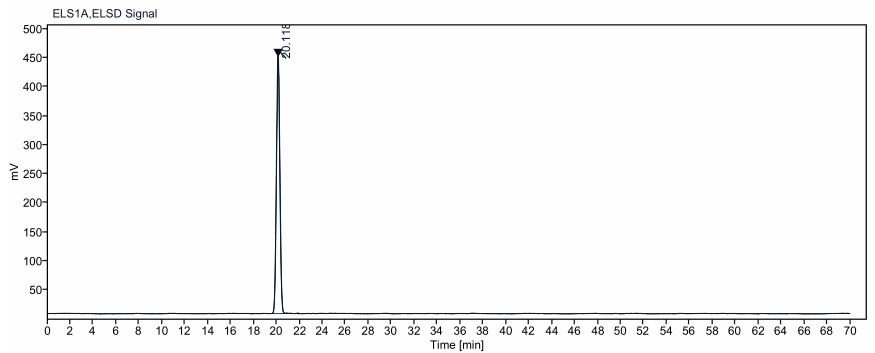
**

1. **LLP**

**
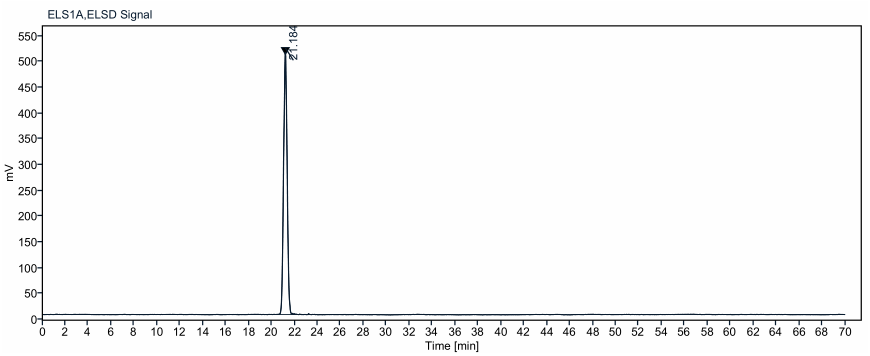
**

1. **OOL**

**
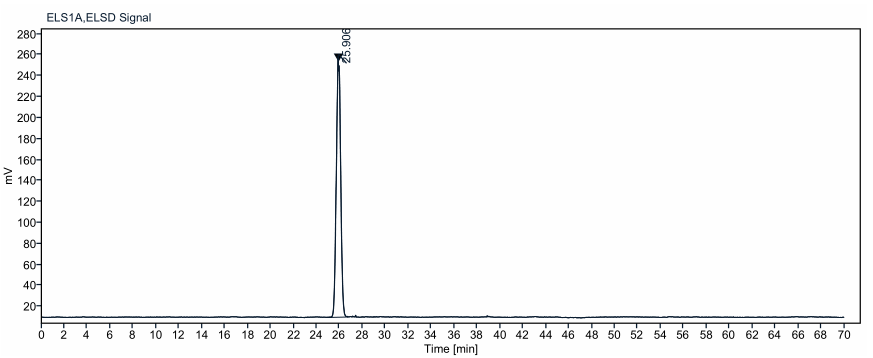
**

1. **POL**

**
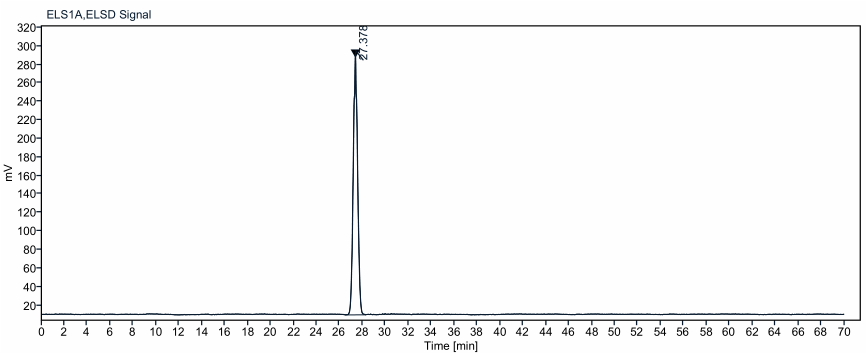
**

1. **OOO**

**
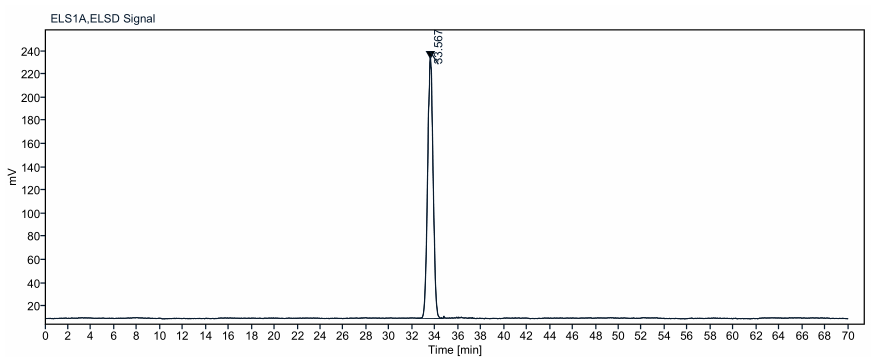
**

1. **OOP**

**
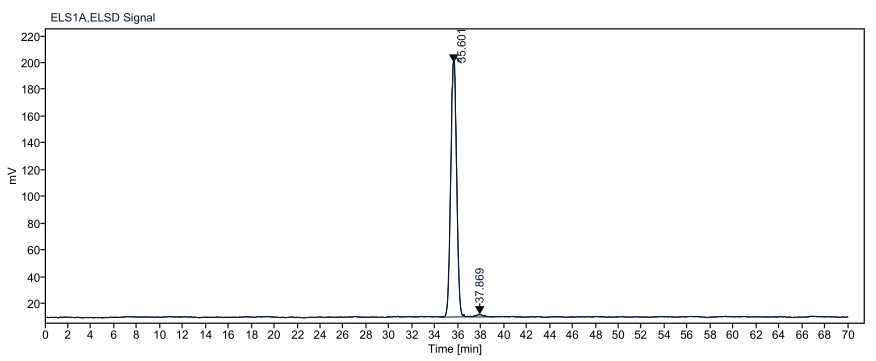
**

1. **POP**

**
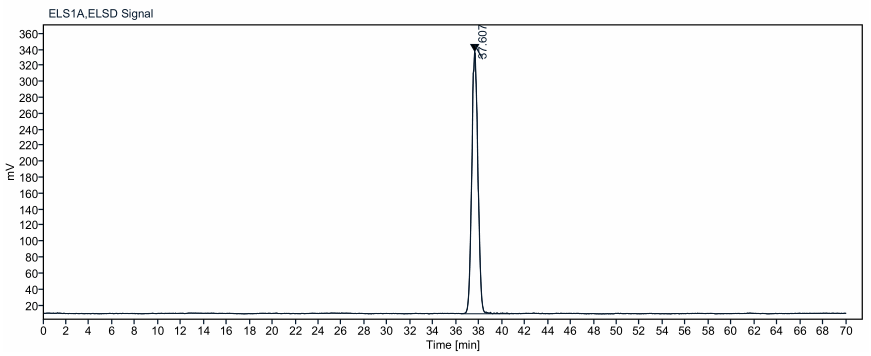
**

**（9）OOS**

**
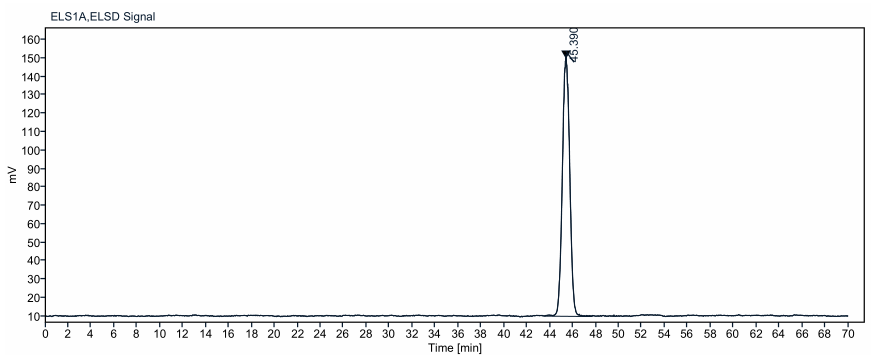
**
